# *TNNT2* Mutation Affects the Progression of Dilated Cardiomyopathy in Children

**DOI:** 10.1016/j.jacasi.2026.05.038

**Published:** 2026-07-23

**Authors:** Shun Liu, Han Mo, Xiao Chen, Hao Cui, Yiqi Zhao, Yifan Wang, Qian Zhao, Zhe Sun, Jun Yan, Ningning Zhang, Xiumeng Hua, Jiangping Song

**Affiliations:** a Department of Cardiac Surgery, Fuwai Hospital, National Center for Cardiovascular Diseases, Chinese Academy of Medical Sciences and Peking Union Medical College, Beijing, China; b Department of Cardiovascular Surgery, Zhongshan Hospital, Fudan University, Shanghai, China; c Shenzhen Key Laboratory of Cardiovascular Disease, Fuwai Hospital, Chinese Academy of Medical Sciences, Shenzhen, China; d State Key Laboratory of Cardiovascular Disease, Fuwai Hospital, National Center for Cardiovascular Diseases, Chinese Academy of Medical Sciences and Peking Union Medical College, Beijing, China; e Beijing Key Laboratory of Xenotransplantation, Fuwai Hospital, National Centre for Cardiovascular Disease, Chinese Academy of Medical Sciences and Peking Union Medical College, Beijing, China; f Department of Cardiac Surgery, Fuwai Yunnan Hospital, Chinese Academy of Medical Sciences, Affiliated Cardiovascular Hospital of Kunming Medical University, Kunming, China

**Keywords:** cardiomyopathy, children, genetic mutations, heart transplantation, whole exome sequencing

## Abstract

**Background:**

Genetic mutations play an important role in pediatric cardiomyopathy. However, whether different genetic factors contribute to the rate of disease progression remains unclear.

**Objectives:**

The authors aimed to explore the role of genetic mutations in disease progression in pediatric cardiomyopathy.

**Methods:**

We collected basic clinical data on 67 children and 166 adults who underwent heart transplantation for cardiomyopathy, and conducted whole-exome sequencing and standard pathology examinations on these patients. We then compared the prevalence of pathogenic genes and their occurring in the same type of myocardium between children and adult heart transplantation recipients, with a focus on the age of onset and duration of disease.

**Results:**

Of 67 children, 33 (49.3%; 95% CI: 32.2%-55.3%) carried mutation genes. The proportion of mutations varied significantly among different types of cardiomyopathies (*P* = 0.003). The median [IQR] time from diagnosis to transplant was significantly shorter in children compared to adults in cases of dilated cardiomyopathy (DCM) (12 [1-23] vs 103 [5-201] months, *P* < 0.001), hypertrophic cardiomyopathy (32 [14.5-46.5] vs 234 [183-285] months, *P* = 0.002) and arrhythmogenic cardiomyopathy (28 [14.5-41.5] vs 127 [86-168] months, *P* < 0.001). Children with DCM were more likely to carry the *TNNT2* mutation compared to adults with DCM (*P* = 0.001), and heart failure progressed more rapidly in children with this mutation versus adults (19 vs 128 months, *P* = 0.002). The most common mutation site in these children was p.K220del.

**Conclusions:**

*TNNT2* mutations were associated with shorter time from diagnosis to transplantation in children with DCM. These findings suggest that early genetic evaluation may aid in risk stratification and inform clinical management in pediatric cardiomyopathy.

Pediatric cardiomyopathy is an inherited condition characterized by diverse phenotypes and clinical heterogeneity. The clinical presentation can range from mild cardiac involvement to severe cardiac dysfunction, ultimately progressing to advanced heart failure (HF) and heart transplantation (HTx).^1^ Four types of cardiomyopathies are particularly prevalent in children, with high morbidity and mortality rates: hypertrophic cardiomyopathy (HCM), dilated cardiomyopathy (DCM), arrhythmogenic cardiomyopathy (ACM), and restrictive cardiomyopathy (RCM).^2^ With advances in genetic testing technology, increasing evidence suggests that genetic variants, including both pathogenic and potentially pathogenic mutations, may play a key role in the development of pediatric cardiomyopathy.^3^^,^^4^ Studies have shown that more than half of children with DCM and HCM carry pathogenic mutations.^5^^,^^6^ Current consensus guidelines recommend genetic testing for pediatric cardiomyopathy patients and regular cardiac monitoring for family members who carry genetic variants.^7^ However, research on the genetics of pediatric cardiomyopathy remains limited, largely because of barriers such as varying socioeconomic levels and perceptions of genetic testing.^7^

Major studies on pediatric cardiomyopathy have primarily focused on identifying gene mutations for diagnostic purposes, with limited research addressing the role of genetic mutation in end-stage HF. A recent study on pediatric HCM found that a higher proportion of pathogenic gene variants and variants of unknown significance were linked to poorer prognosis.^8^ Similarly, another study on pediatric cardiomyopathy prognosis also highlighted the significant impact of genetic mutations on patient outcomes.^9^ These findings suggest that genetic mutations play a critical role in the prognostic landscape of pediatric cardiomyopathy. Large-scale studies have identified 47 risk loci associated with HF.^10^ However, the precise contribution of genetic variants related to cardiomyopathies in predicting the progression to end-stage HF in children remains insufficiently understood.

HTx remains a highly effective treatment for end-stage HF. Nearly 14,000 pediatric HTx cases have been reported in the International Society for Heart and Lung Transplantation (ISHLT) Registry.^11^ Given the unique aspects of children's growth and development, improving prognosis in this population is especially crucial. Research has shown that children with HCM face a risk of HTx that is twice as high as that of adults. Additionally, there are significant differences in clinical features, genotyping, and cardiovascular outcomes between pediatric and adult HCM patients.^12^ Cardiomyopathy-induced HF in children is often more severe and fatal than in adults.^13^ Children with mitochondrial cardiomyopathy also experience a higher rate of post-HTx morbidity, necessitating more cautious management in this group.^14^ Although the genetic causes of cardiomyopathy are well documented, their specific impact on the progression to end-stage HF requiring HTx remains poorly understood. The role of genetic mutations in accelerating the progression to HF in children, ultimately leading to HTx, has yet to be fully elucidated.

This study focused on analyzing whole-exome sequencing (WES) data from a transplant cohort of pediatric patients with end-stage HF due to cardiomyopathy. It examined the types and proportions of genetic mutations and compared these findings with an adult cardiomyopathy HTx cohort to explore the role of mutations in HF associated with pediatric cardiomyopathy.

## Materials and Methods

### Study design

Our team conducted this retrospective cohort study in our center. It included children younger than 18 years who underwent HTx between October 2004 and July 2023. For comparison, we chose a cohort of adults who underwent HTx from the same time. All patients underwent WES. Clinical data were collected by reviewing electronic medical records. The study protocol and data publication were approved by the Institutional Review Board (IRB) of Fuwai Hospital by the Declaration of Helsinki (ethical approval number: 2022-1727, approval date: October 13, 2022). All patients or their families provided written informed consent.

### Patients

The inclusion criteria for this study were primary cardiomyopathy diagnosed before the age of 18 years and phenotypes of DCM, HCM, ACM, and RCM. All HTx were performed at our center. Heart functions were measured using echocardiography and cardiac magnetic resonance imaging. Children with other secondary cardiomyopathies, myocarditis, cardiotoxic exposure, or other congenital heart diseases were excluded.

The time of initial diagnosis was the first time diagnosed according to the diagnostic criteria for cardiomyopathy. Diagnostic criteria were defined according to American Heart Association guidelines.^15^ DCM is based on left ventricular end-diastolic diameter and left ventricular end-systolic diameter *z*-scores >2 on either side of the body surface area.^1^ The primary diagnostic criterion for HCM is the maximum diastolic septal or left ventricular free wall thickness; published criteria in adults use 15 mm as the definitive threshold. Adjusting for body size is mandatory in children. The primary diagnostic criterion for RCM is impaired myocardial compliance. This abnormal compliance of the ventricles is caused by abnormalities in the myocyte or the intercellular matrix, such as interstitial infiltration or fibrosis.^16^ The diagnosis of ACM is established by a point score according to the revised 2010 Task Force criteria using imaging modalities, biopsy evidence of fibrofatty replacement, electrocardiographic abnormalities, ventricular arrhythmias, and positive family history, including identified genetic mutations.^17^

### Genetic testing and variant analysis

Genomic DNA was extracted from peripheral blood following the manufacturer's instructions (Blood DNA Extraction Kit, Enriching Biotechnology). WES was performed using captured next-generation sequencing on the Illumina 2500 platform (Illumina). Sequencing reads were aligned to the human reference genome (GRCh37/hg19) using the Burrows-Wheeler Aligner, and variant calling was performed using the Genome Analysis Toolkit pipeline. Sequencing quality was rigorously controlled, achieving an average on-target sequencing depth of approximately 100×, with >98% of the targeted regions covered at a depth of ≥20×.

To ensure strict genotype-phenotype correlation and to exclude genetic variants unrelated to the patients' clinical presentation, we adopted a targeted analysis strategy on the acquired WES data. Downstream bioinformatics filtering and interpretation were restricted exclusively to a predefined panel of 189 genes (Supplemental Table 1) definitively associated with inherited cardiovascular diseases and cardiomyopathies.

Variants within these 189 genes were annotated according to the following criteria: 1) variants with a minor allele frequency (MAF) of <1% in the 1000 Genomes Project (1000g_all),^18^ esp6500siv2_all,^19^ and gnomAD data (gnomAD_ALL and gnomAD_EAS)^20^ were included. 2) Only single-nucleotide variants (SNVs) occurring in exons or splice sites (splicing junction ±10 bp) were further analyzed, as we were interested in amino acid changes. 3) Synonymous SNVs which do not result in amino acid alterations, predicted by dbscSNV, were discarded. Similarly, small, non-frameshift (<10 bp) indels in repeat regions defined by RepeatMasker were excluded. 4) Variations were screened according to scores of SIFT,^21^ Polyphen,^22^ MutationTaster,^23^ and CADD^24^ software. The potentially deleterious variants were retained if more than half of the scores from these 4 software tools supported their harmfulness.^25^ Sites (>2 bp) that did not affect alternative splicing were removed. 5) For *TTN* variants, only truncated variants were considered for pathogenesis analysis.

To improve the prediction of the harmfulness of genetic variations, the classification system established by the American College of Medical Genetics and Genomics (ACMG) was applied. This system categorizes variations into the following categories: pathogenic, likely pathogenic, variants of unknown significance, likely benign, or benign.^26^ A proband is considered to have a positive result if it carries a mutation classified as either pathogenic or likely pathogenic. Detailed variant characteristics, including specific Human Genome Variation Society (HGVS) nomenclature, population frequencies, and the corresponding ACMG interpretation codes, are summarized in Supplemental Table 2.

### Pathological examination

Each explanted heart with a clinical diagnosis of primary cardiomyopathy was examined according to international statements.^27^ Six representative specimens of the midventricular level were collected for histological examination. Two pathologists, blinded to clinical and genetic data, performed a microscopic examination of the specimens. Hematoxylin and eosin and Masson's staining were used to compare the distribution of cardiomyocytes, degree of fibrosis, and fat in the tissues. The slides were scanned for digital images. All pathological images obtained using ZEN microscopy software (version 2.3; Zeiss) were viewed and analyzed, and structures and graphic layouts were drawn using Adobe Illustrator CC 2018 (Adobe).

### Statistical analysis

Descriptive statistics were reported as count (%), mean ± standard deviation, or median (IQR). Categorical variables were compared using Fisher's exact test. For comparisons between 2 independent groups, continuous variables were compared using the Student's *t*-test for normally distributed data (assessed by Shapiro-Wilk test) or the Mann-Whitney *U* test for non-normally distributed data. For comparisons involving 3 or more independent groups, the Kruskal-Wallis test was used. This nonparametric test was selected given the presence of small subgroup sample sizes and the lack of normality in several continuous variables. The Kruskal-Wallis test does not assume normality or homogeneity of variances, making it appropriate for our data structure. When the Kruskal-Wallis test indicated a statistically significant difference (*P* < 0.05), post hoc pairwise comparisons were performed using Dunn's test with Bonferroni correction for multiple comparisons. These post hoc results are referenced in the table footnotes where applicable. All continuous variables are presented as median (IQR) in the tables, consistent with the use of nonparametric methods.

Time-to-event curves for the interval from initial diagnosis to HTx were estimated using the Kaplan-Meier method. Between-group comparisons were performed using the log-rank test. As all patients ultimately underwent HTx during the study period, the analysis focused on comparing the distribution of time to HTx rather than traditional survival with censoring. For exploratory univariate analyses, we estimated the association between selected clinical variables and the time from diagnosis to HTx using the Cox proportional hazards model. The dependent variable was the time to HTx, with all patients experiencing the event (HTx) during follow-up. Statistical significance (*P* < 0.05) was accepted within the 95% CI. For all reported proportions (percentages), 95% CIs were calculated using the Wilson score method for samples with n ≥ 30 and the Clopper-Pearson exact method for samples with n < 30. For Kaplan-Meier estimates of median time to HTx, 95% CIs were calculated using the Brookmeyer-Crowley method. For the accelerated failure time (AFT) model, 95% CIs for time ratios were derived from model-based standard errors using the delta method. All confidence intervals are reported alongside point estimates in the text, tables, and figures. To account for multiple comparisons across multiple genes, we applied the Benjamini-Hochberg false discovery rate (FDR) correction to all exploratory analyses involving gene distribution comparisons. We constructed an AFT model with log-normal distribution. All 49 pediatric DCM patients underwent HTx (100% event rate). The Cox model was designed for censored data; the AFT model is more appropriate for complete event datasets, as it directly models the event time distribution. The AFT model provides time ratios, which directly quantify “how much faster” *TNNT2* carriers progress compared to noncarriers (time ratio = 0.38 means 2.6 times faster). The Cox model provides hazard ratios, which are less intuitive for time acceleration interpretation. The Cox model requires the proportional hazards assumption. In our data, the proportional hazards assumption was violated for *TNNT2* (global Schoenfeld test *P* = 0.031). The AFT model does not require this assumption. We fitted 3 candidate distributions (Weibull, log-logistic, log-normal) and selected the log-normal distribution based on the lowest Akaike information criterion (287.3 vs 291.6 and 290.1). All statistical analysis was performed with SPSS Statistics 24.0 software (IBM Corp). Data were plotted using the GraphPad Prism program (version 8.0; GraphPad).

## Results

### Patient clinical characteristics

A total of 67 children were included in the final analysis during the study period (Figure 1). Of these, 49 (73.13%) had DCM, 4 (5.97%) had HCM, 3 (4.48%) had RCM, and 11 (16.42%) had ACM (Figure 2A and Table 1). There were no significant differences in age at surgery (Kruskal-Wallis *P* = 0.382) or sex (Fisher's exact *P* = 0.870) across the different types of cardiomyopathies (Table 1). We observed significant variability in the time from diagnosis to HTx (*P* = 0.043; Table 1 and Supplemental Figure 1). Children with DCM had the shortest time to HTx, with a median of 12 months (Table 1). Additionally, preoperative left ventricular ejection fraction (LVEF) (*P* < 0.001) and left ventricular internal diameter at end-diastole (LVIDd) (*P* < 0.001) were significantly different in children with DCM compared to those with other types of cardiomyopathies (Table 1, Figure 2B). This is consistent with the characteristic progression of DCM, where children may rapidly progress to end-stage HF, necessitating early HTx.

**Figure 1:**
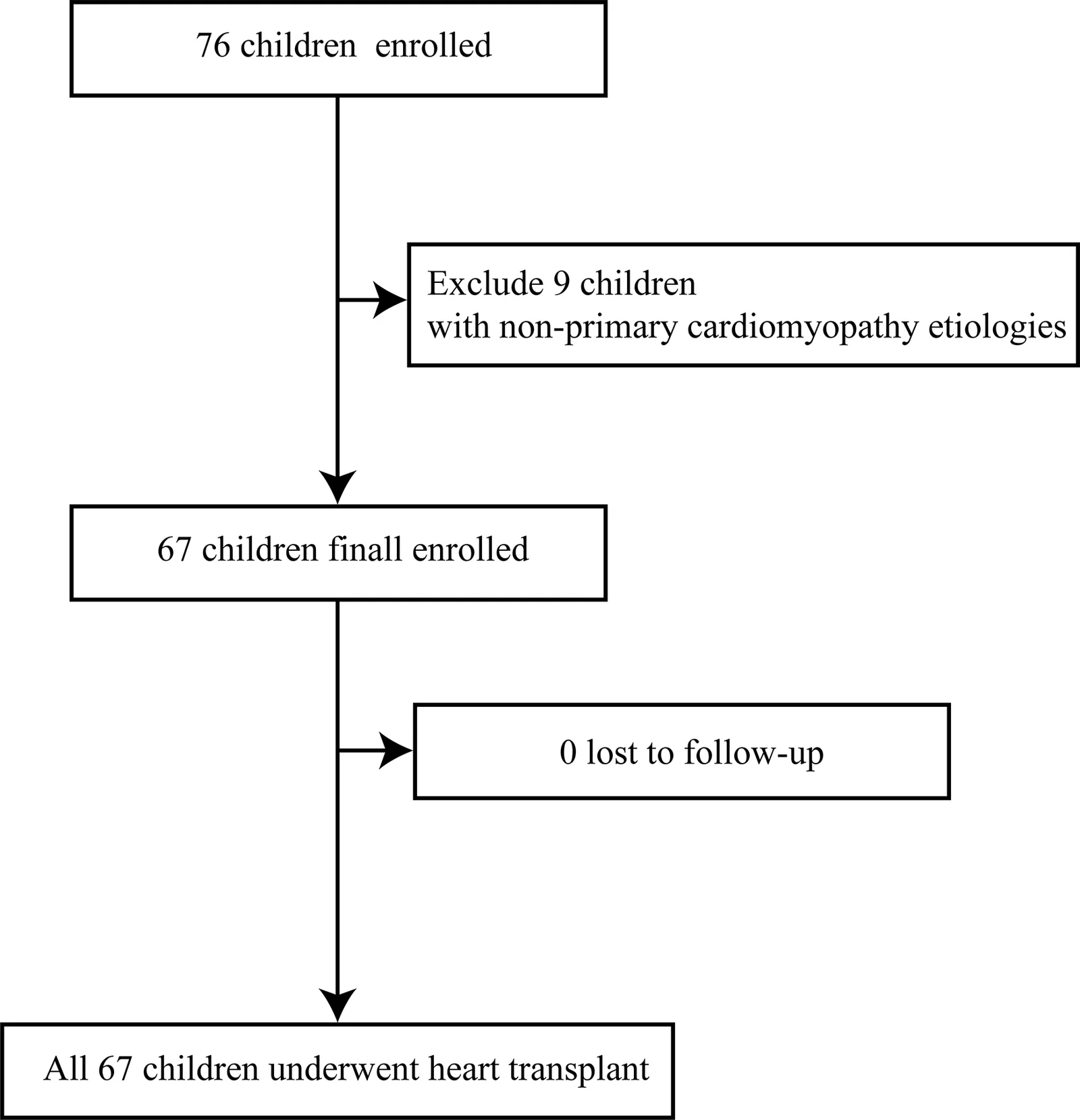
Study Flow Diagram of Pediatric Patient Selection Out of 76 children undergoing heart transplantation for cardiomyopathy between October 2004 and July 2023, 9 were excluded because of nonprimary cardiomyopathy etiologies (myocarditis, cardiotoxic exposure, or congenital heart disease). The final analysis included 67 children with primary dilated, hypertrophic, restrictive, or arrhythmogenic cardiomyopathy. All 67 patients underwent whole-exome sequencing and were followed up until transplantation; none were lost to follow-up. This cohort formed the basis for comparing genetic mutations, clinical characteristics, and time-to-event outcomes with an adult transplantation cohort.

**Figure 2:**
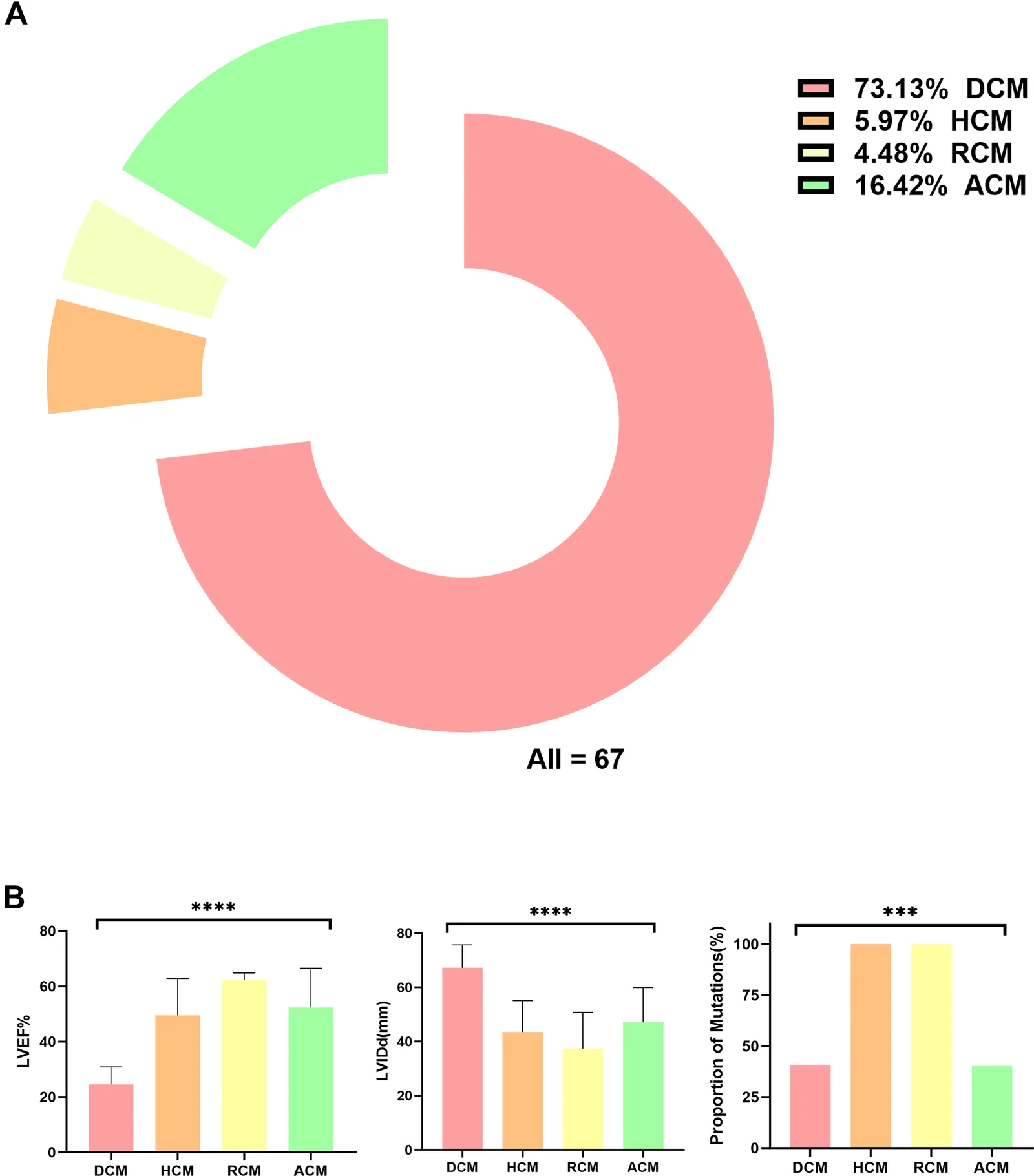
Distribution and Clinical Characteristics of Pediatric End-Stage Cardiomyopathies (A) Pie chart showing the proportion of 67 children who underwent heart transplantation (HTx) for dilated (73.1%), hypertrophic (6.0%), restrictive (4.5%), and arrhythmogenic (16.4%) cardiomyopathy. (B) Box plots comparing left ventricular ejection fraction (LVEF), left ventricular internal diameter at end-diastole (LVIDd), and proportion of genetic mutations across the 4 groups. Kruskal-Wallis test was used for continuous variables; Fisher's exact test for proportions. Error bars indicate 95% confidence intervals. Pediatric patients with dilated cardiomyopathy had significantly lower LVEF, larger LVIDd, and similar mutation prevalence compared to other types (all *P* < 0.05). These findings highlight dilated cardiomyopathy as the leading cause of pediatric HTx and confirm phenotypic diversity. ACM = arrhythmogenic cardiomyopathy; DCM = dilated cardiomyopathy; HCM = hypertrophic cardiomyopathy; RCM = restrictive cardiomyopathy. ∗∗∗*P* < 0.05. ∗∗∗∗*P* < 0.01.

**Table 1:** Clinical Characteristics of Explant Tissues From Pediatric Heart Transplantation Recipients

	Children (N = 67)				*P* Value
	DCM (n = 49)	HCM (n = 4)	RCM (n = 3)	ACM (n = 11)	
Clinical characteristics					
Age at surgery, y	15 (13-17)	16 (15-17)	15 (14-16)	14 (13-15)	0.382
Time to HTx, mo^a^	12 (1-23)	32 (14.5-46.5)	38 (28-48)	28 (14.5-41.5)	**0.043**
Male/female, n	34/15	2/2	2/1	8/3	0.870
Comorbidities					
Type 2 diabetes	1 (2.0%; 95% CI: 0.1-10.6)	0 (0.0%; 95% CI: 0-60.2)	0 (0.0%; 95% CI: 0-70.8)	0 (0.0%; 95% CI: 0-28.5)	0.949
Hypertension	0 (0.0%; 95% CI: 0-7.3)	0 (0.0%; 95% CI: 0-60.2)	0 (0.0%; 95% CI: 0-70.8)	0 (0.0%; 95% CI: 0-28.5)	1.000
Kidney disease	2 (4.0%; 95% CI: 0.5-14.0)	0 (0.0%; 95% CI: 0-60.2)	0 (0.0%; 95% CI: 0-70.8)	0 (0.0%; 95% CI: 0-28.5)	0.868
Pulmonary hypertension	25 (51.0%; 95% CI: 36.6-65.4)	0 (0.0%; 95% CI: 0-60.2)	1 (33.3%; 95% CI: 0.8-90.6)	1 (9.1%; 95% CI: 0.2-41.3)	**0.030**
Arrhythmias					
AF/flutter	1 (2.0%; 95% CI: 0.1-10.6)	1 (25.0%; 95% CI: 0.6-80.6)	0 (0.0%; 95% CI: 0-70.8)	0 (0.0%; 95% CI: 0-28.5)	0.063
VT/VF	9 (18.4%; 95% CI: 8.8-32.0)	1 (25.0%; 95% CI: 0.6-80.6)	0 (0.0%; 95% CI: 0-70.8)	6 (54.5%; 95% CI: 23.4-83.3)	0.099
Echocardiography					
LVPWT, mm	8 (7.5-8.5)	9.5 (7-12)	10 (6-14)	8 (7.5-8.5)	**0.001**
LVEF, %	25 (20-30)	54 (41.5-66.5)	53 (51-55)	50 (34.5-65.5)	**<0.001**
LVIDd, mm	65 (59.5-70.5)	40.5 (30-51)	43 (22-64)	47 (34-60)	**<0.001**
Devices					
ICD	2 (4.1%; 95% CI: 0.5-14.0)	0 (0.0%; 95% CI: 0-60.2)	0 (0.0%; 95% CI: 0-70.8)	1 (9.1%; 95% CI: 0.2-41.3)	0.837
CRT-D	0 (0.0%; 95% CI: 0-7.3)	0 (0.0%; 95% CI: 0-60.2)	0 (0.0%; 95% CI: 0-70.8)	0 (0.0%; 95% CI: 0-28.5)	1.000
VAD	0 (0.0%; 95% CI: 0-7.3)	0 (0.0%; 95% CI: 0-60.2)	0 (0.0%; 95% CI: 0-70.8)	0 (0.0%; 95% CI: 0-28.5)	1.000
Medications					
ACEi/ARB	19 (38.8%; 95% CI: 25.2-53.8)	3 (75.0%; 95% CI: 19.4-99.4)	0 (0.0%; 95% CI: 0-70.8)	5 (45.5%; 95% CI: 16.8-76.6)	0.250
Beta-blocker	29 (59.2%; 95% CI: 44.4-72.8)	2 (50.0%; 95% CI: 6.8-93.2)	1 (33.3%; 95% CI: 0.8-90.6)	9 (81.8%; 95% CI: 48.2-97.7)	0.369
Amiodarone	3 (6.1%; 95% CI: 1.3-16.9)	1 (25.0%; 95% CI: 0.6-80.6)	0 (0.0%; 95% CI: 0-70.8)	0 (0.0%; 95% CI: 0-28.5)	0.337
MRA	44 (89.8%; 95% CI: 78.0-96.1)	4 (100.0%; 95% CI: 39.8-100)	3 (100%; 95% CI: 29.2-100)	10 (90.9%; 95% CI: 58.7-99.8)	0.862
Genetic mutation	20 (40.8%; 95% CI: 27.0-55.8)	4 (100.0%; 95% CI: 39.8-100)	3 (100.0%; 95% CI: 29.2-100)	5 (45.5%; 95% CI: 16.8-76.6)	**0.033**

Categorical variables are presented as n (%), and continuous variables are presented as median (IQR) unless otherwise indicated. Comparisons across the 4 cardiomyopathy types (DCM, HCM, RCM, ACM) were performed using the Kruskal-Wallis test, as indicated in the rightmost column (*P* value). Categorical variables were compared using Fisher's exact test. Proportions are presented as n (%); 95% CIs were calculated using the Wilson score method for n ≥ 30 and the Clopper-Pearson exact method for n <30. **Bolded***P* values indicate statistically significant differences between groups.

ACEi = angiotensin-converting enzyme inhibitor; ACM = arrhythmogenic cardiomyopathy; AF = atrial fibrillation; ARB = angiotensin receptor blockers; CRT-D = cardiac resynchronization therapy with defibrillator; DCM = dilated cardiomyopathy; HCM = hypertrophic cardiomyopathy; ICD = implantable cardioverter-defibrillator; LVEF = left ventricular ejection fraction; LVIDd = left ventricular internal dimension in diastole; LVPWT = left ventricular posterior wall thickness; MRA = mineralocorticoid receptor antagonists; RCM = restrictive cardiomyopathy; VAD = ventricular assist device; VT/VF = ventricular tachycardia/ventricular fibrillation.

a Time to HTx indicates time from initial diagnosis to heart transplantation in months.

A total of 166 adults underwent HTx at our center in the same time. The median age at HTx was 50 years (IQR: 31-69) for those with DCM, 35 years (IQR: 20-50) for those with HCM, 46 years (IQR: 30-62) for those with RCM, and 43 years (IQR: 31-55) for those with ACM. Similar to the pediatric cohort, we observed variability the time from diagnosis to HTx in different types of cardiomyopathies (*P* < 0.001; Table 2 and Supplemental Figure 2). Notably, the shortest time was seen in patients with RCM (48 months; Table 2). Regarding arrhythmic events, there were significant differences between the cardiomyopathy types (*P* = 0.002; Table 2). Ventricular fibrillation was most common in patients with ACM (17 of 24, 70.8%; Table 2), which is consistent with the arrhythmic characteristics of ACM. Additionally, patients with DCM differed significantly from other types of cardiomyopathies in terms of LVEF (*P* < 0.001) and LVIDd (*P* < 0.001), similar to what we observed in the pediatric cohort (Table 2).

**Table 2:** Clinical Characteristics of Explant Tissues From Adult Heart Transplantation Recipients

Characteristic	Adults (N = 166)				*P* Value
	DCM (n = 120)	HCM (n = 10)	RCM (n = 10)	ACM (n = 24)	
Clinical characteristics					
Age at surgery, y	50 (31-69)	35 (20-50)	46 (30-62)	43 (31-55)	0.096
Time to HTx, mo^a^	103 (5-201)	234 (183-285)	48 (13-83)	127 (86-168)	**<0.001**
Male/female, n	94/26	8/2	6/4	18/6	0.605
Comorbidities					
Type 2 diabetes	23 (19.2%; 95% CI: 12.6-27.5)	2 (20.0%; 95% CI: 2.5-55.6)	0 (0; 95% CI: 0-30.8)	1 (4.2%; 95% CI: 0.1-21.1)	0.141
Hypertension	18 (15.0%; 95% CI: 9.2-22.7)	2 (20.0%; 95% CI: 2.5-55.6)	0 (0; 95% CI: 0-30.8)	3 (12.5%; 95% CI: 2.7-32.4)	0.558
Kidney disease	15 (12.5%; 95% CI: 7.2-19.9)	1 (10.0%; 95% CI: 0.3-44.5)	2 (20.0%; 95% CI: 2.5-55.6)	2 (8.3%; 95% CI: 1.0-27.0)	0.811
Pulmonary hypertension	69 (57.5%; 95% CI: 48.2-66.4)	5 (50.0%; 95% CI: 18.7-81.3)	3 (30.0%; 95% CI: 6.7-65.2)	8 (33.3%; 95% CI: 15.6-55.3)	0.080
Arrhythmias					
AF/flutter	55 (45.8%; 95% CI: 36.7-55.2)	6 (60.0%; 95% CI: 26.2-87.8)	5 (50.0%; 95% CI: 18.7-81.3)	11 (45.8%; 95% CI: 25.6-67.2)	0.851
VT/VF	64 (53.3%; 95% CI: 44.0-62.5)	2 (20.0%; 95% CI: 2.5-55.6)	1 (10.0%; 95% CI: 0.3-44.5)	17 (70.8%; 95% CI: 48.9-87.4)	**0.002**
Echocardiography					
LVPWT, mm	9.0 (8.0-10.0)	9.5 (6.5-12.5)	9.0 (8.0-10.0)	9.0 (8.0-10.0)	**0.030**
LVEF, %	25 (15-35)	32 (27-37)	44 (19-69)	34 (30-38)	**<0.001**
LVIDd, mm	75.0 (59.0-91.0)	60.5 (37.5-82.5)	46.5 (42.5-50.5)	68.5 (49.5-87.5)	**<0.001**
Devices					
ICD	35 (29.2%; 95% CI: 21.2-38.2)	0 (0; 95% CI: 0-30.8)	3 (30.0%; 95% CI: 6.7-65.2)	13 (54.2%; 95% CI: 32.8-74.4)	**0.014**
CRT-D	22 (18.3%)	0 (0; 95% CI: 0-30.8)	0 (0; 95% CI: 0-30.8)	10 (41.7%; 95% CI: 22.1-63.4)	**0.006**
VAD	2 (1.7%; 95% CI: 0.2-5.9)	0 (0; 95% CI: 0-30.8)	0 (0; 95% CI: 0-30.8)	0 (0; 95% CI: 0-14.2)	0.993
Medications					
ACEi/ARB	56 (46.7%; 95% CI: 37.5-56.0)	2 (20.0%; 95% CI: 2.5-55.6)	2 (20.0%; 95% CI: 2.5-55.6)	7 (29.2%; 95% CI: 12.6-51.1)	0.085
Beta-blocker	102 (85.0%; 95% CI: 77.3-90.7)	8 (80.0%; 95% CI: 44.4-97.5)	6 (60.0%; 95% CI: 26.2-87.8)	12 (50.0%; 95% CI: 29.1-70.9)	**0.001**
Amiodarone	26 (21.7%; 95% CI: 14.8-30.0)	3 (30.0%; 95% CI: 6.7-65.2)	1 (10.0%; 95% CI: 0.3-44.5)	16 (66.7%; 95% CI: 44.7-84.4)	**<0.001**
MRA	118 (98.3%; 95% CI: 94.2-99.8)	10 (100.0%; 95% CI: 69.2-100)	7 (70.0%; 95% CI: 34.8-93.3)	24 (100.0%; 95% CI: 85.8-100)	**<0.001**
Genetic mutation	39 (32.5%; 95% CI: 24.2-41.8)	2 (20.0%; 95% CI: 2.5-55.6)	7 (70.0%; 95% CI: 34.8-93.3)	17 (70.8%; 95% CI: 48.9-87.4)	**<0.001**

Categorical variables are presented as n (%), and continuous variables are presented as median (IQR) unless otherwise indicated. Comparisons across the 4 cardiomyopathy types (DCM, HCM, RCM, ACM) were performed using the Kruskal-Wallis test, as indicated in the rightmost column (*P*-value). Categorical variables were compared using Fisher's exact test. **Bolded***P* values indicate statistically significant differences between groups.

Abbreviations as in Table 1.

a Time to HTx indicates time from initial diagnosis to heart transplantation in months.

### Time from diagnosis to HTx was significantly shorter in pediatric cardiomyopathy

We further compared the basic data of patients with the same type of cardiomyopathy between children and adults. As illustrated in the Kaplan-Meier curves, the cumulative incidence of HTx over time differed significantly between children and adults. Children with DCM reached the endpoint of HTx significantly earlier than adults (median: 12 months [95% CI: 8-18] vs 103 months [95% CI: 81-142]; log-rank *P* < 0.001; Table 3 and Figure 3A). Among adults with DCM, 55 of 120 (45.8%) had atrial fibrillation, and 64 (53.3%) had ventricular fibrillation. Adults were more likely to experience malignant arrhythmic events compared to children (*P* < 0.001; Table 3). In HCM and ACM, we also observed a significantly shorter time from diagnosis to HTx in children compared to adults (*P* < 0.05; Table 3 and Figure 3B and 3D). Regarding arrhythmic events, the incidence of atrial fibrillation was higher in adults with ACM (11 of 24, 45.8%) compared to children (0 of 11, 0%) (*P* = 0.007; Table 3). Overall, adults had higher rates of arrhythmic events in both DCM and ACM.

**Table 3:** Clinical Characteristics of Heart Transplantation Recipients Between Children and Adults

	DCM			HCM			RCM			ACM		
	Adults (n = 120)	Children (n = 49)	*P* Value	Adults (n = 10)	Children (n = 4)	*P* Value	Adults (n = 10)	Children (n = 3)	*P* Value	Adults (n = 24)	Children (n = 11)	*P* Value
Clinical characteristics												
Age at surgery, y	50 (31-69)	15 (13-17)	**<0.001**	35 (20-50)	16 (15-17)	**0.002**	46 (30-62)	15 (14-16)	**0.007**	43 (31-55)	14 (13-15)	**<0.001**
Time to HTx, mo^a^	103 (5-201)	12 (1-23)	**<0.001**	234 (183-285)	32 (14.5-46.5)	**0.002**	48 (13-83)	38 (28-48)	0.692	127 (86-168)	28 (14.5-41.5)	**<0.001**
Sex, male/female, n	94/26	34/15	0.218	8/2	2/2	0.262	6/4	2/1	0.835	18/6	8/3	0.886
Comorbidities												
Type 2 diabetes	23 (19.2%)	1 (2.0%)	**0.004**	2 (20.0%)	0 (0.0%)	0.334	0 (0)	0 (0.0%)	1.000	1 (4.2%)	0 (0.0%)	0.492
Hypertension	18 (15.0%)	0 (0.0%)	**0.004**	2 (20.0%)	0 (0.0%)	0.334	0 (0)	0 (0.0%)	1.000	3 (12.5%)	0 (0.0%)	0.220
Kidney disease	15 (12.5%)	2 (4.0%)	0.099	1 (10.0%)	0 (0.0%)	0.512	2 (20.0%)	0 (0.0%)	0.400	2 (8.3%)	0 (0.0%)	0.324
Pulmonary hypertension	69 (57.5%)	25 (51.0%)	0.312	5 (50.0%)	0 (0.0%)	0.078	3 (30.0%)	1 (33.3%)	0.913	8 (33.3%)	1 (9.1%)	0.128
Arrhythmias												
AF/flutter	55 (45.8%)	1 (2.0%)	**<0.001**	6 (60.0%)	1 (25.0%)	0.237	5 (50.0%)	0 (0.0%)	0.118	11 (45.8%)	0 (0.0%)	**0.007**
VT/VF	64 (53.3%)	9 (18.4%)	**<0.001**	2 (20.0%)	1 (25.0%)	0.837	1 (10.0%)	0 (0.0%)	0.326	17 (70.8%)	6 (54.5%)	0.346
Echocardiography												
LVPWT, mm	9.0 (8.0-10.0)	8 (7.5-8.5)	**<0.001**	9.5 (6.5-12.5)	9.5 (7-12)	0.839	9.0 (8.0-10.0)	10 (6-14)	0.811	9.0 (8.0-10.0)	8 (7.5-8.5)	**0.005**
LVEF, %	25 (15-35)	25 (20-30)	0.684	32 (27-37)	54 (41.5-66.5)	0.054	44 (19-69)	53 (51-55)	0.217	34 (30-38)	50 (34.5-65.5)	0.224
LVIDd, mm	75.0 (59.0-91.0)	65 (59.5-70.5)	**<0.001**	60.5 (37.5-82.5)	40.5 (30-51)	**0.036**	46.5 (42.5-50.5)	43 (22-64)	0.287	68.5 (49.5-87.5)	47 (34-60)	**0.003**
Devices												
ICD	35 (29.2%)	2 (4.1%)	**<0.001**	0 (0)	0 (0.0%)	1.000	3 (30.0%)	0 (0.0%)	0.279	13 (54.2%)	1 (9.1%)	**0.012**
CRT-D	22 (18.3%)	0 (0.0%)	**0.001**	0 (0)	0 (0.0%)	1.000	0 (0)	0 (0.0%)	1.000	10 (41.7%)	0 (0.0%)	**0.011**
VAD	2 (1.7%)	0 (0.0%)	**0.020**	0 (0)	0 (0.0%)	1.000	0 (0)	0 (0.0%)	1.000	0 (0)	0 (0.0%)	1.000
Medications												
ACEi/ARB	56 (46.7%)	19 (38.8%)	0.349	2 (20.0%)	3 (75.0%)	0.052	2 (20.0%)	0 (0.0%)	0.400	7 (29.2%)	5 (45.5%)	0.346
Beta-blocker	102 (85.0%)	29 (59.2%)	**<0.001**	8 (80.0%)	2 (50.0%)	0.262	6 (60.0%)	1 (33.3%)	0.416	12 (50.0%)	9 (81.8%)	0.074
Amiodarone	26 (21.7%)	3 (6.1%)	**0.015**	26 (21.7%)	1 (25.0%)	0.852	1 (10.0%)	0 (0.0%)	0.569	16 (66.7%)	0 (0.0%)	**<0.001**
MRA	118 (98.3%)	44 (89.8%)	**0.011**	118 (98.3%)	4 (100.0%)	1.000	7 (70.0%)	3 (100%)	0.279	24 (100.0%)	10 (90.9%)	0.134
Genetic mutation	39 (32.5%)	20 (40.8%)	0.303	2 (20.0%)	4 (100.0%)	**0.015**	7 (70.0%)	3 (100.0%)	0.528	17 (70.8%)	5 (45.5%)	0.149

Categorical variables are presented as n (%), and continuous variables are presented as median (IQR) unless otherwise indicated. **Bolded***P* values indicate statistically significant differences between groups.

Abbreviations as in Table 1.

a Time to HTx indicates time from initial diagnosis to heart transplantation in months.

**Figure 3:**
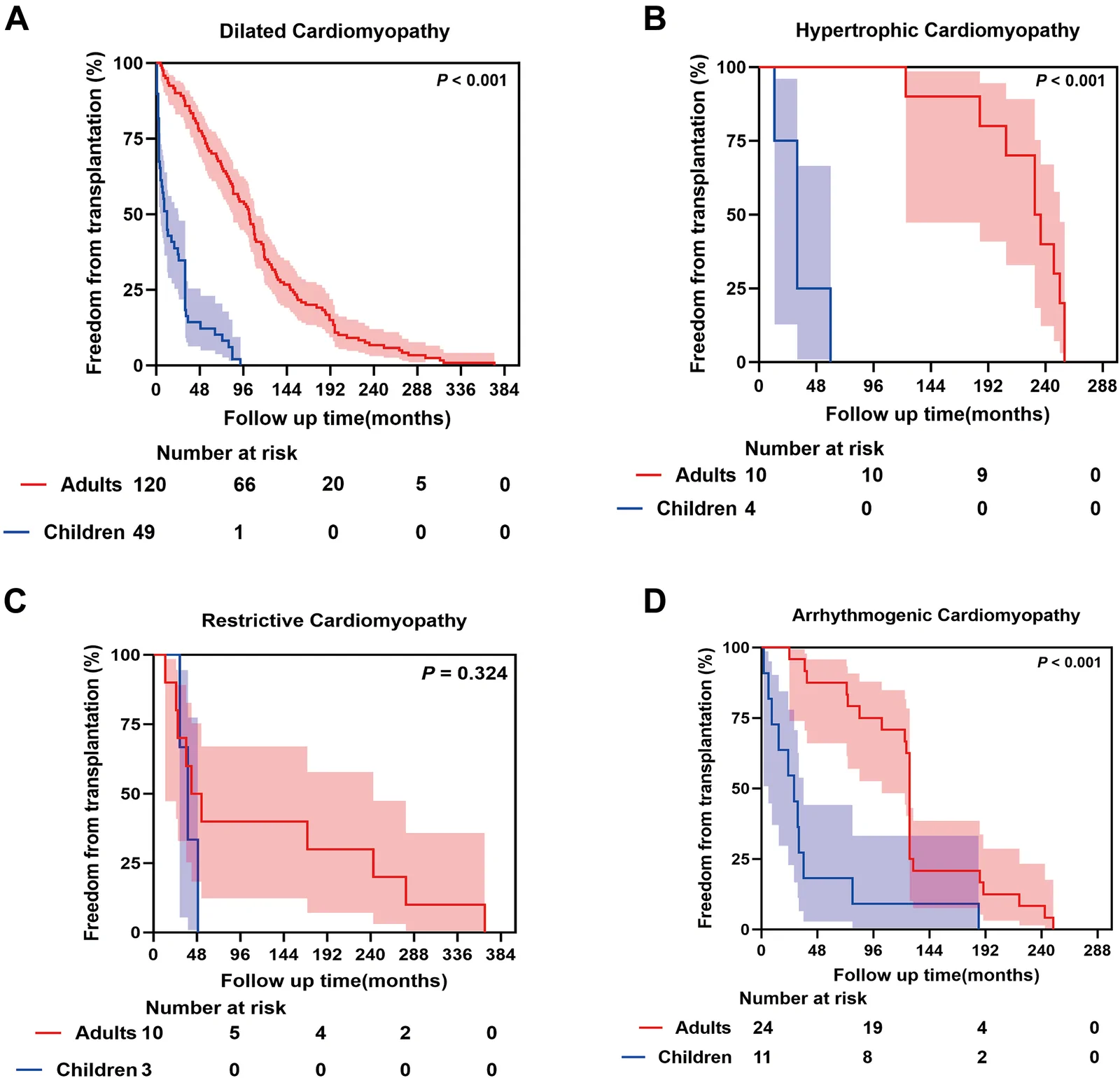
Time From Diagnosis to Heart Transplantation in Children vs Adults Kaplan-Meier curves comparing time to heart transplantation between children (blue) and adults (red) with (A) dilated cardiomyopathy (DCM), (B) hypertrophic cardiomyopathy (HCM), (C) restrictive cardiomyopathy (RCM), and (D) arrhythmogenic cardiomyopathy (ACM). Because all patients underwent transplantation, curves depict cumulative incidence over time, not survival. Shaded areas represent 95% confidence intervals (Brookmeyer-Crowley method). Log-rank *P* values: DCM <0.001, HCM = 0.002, RCM = 0.692, ACM <0.001. Children with DCM, HCM, and ACM underwent transplantation significantly earlier than adults, indicating more rapid disease progression in pediatric populations. Number at risk at each time point is provided below each panel.

### Genetic mutations were associated with shorter time from the diagnosis to HTx in pediatric cardiomyopathy

We performed WES-based targeted analysis of 189 cardiomyopathy-associated genes on all pediatric and adult patients. Among the pediatric patients, 33 of 67 (49.3%) carried pathogenic gene mutations, while 65 of 166 (39.2%) of the adult patients had pathogenic gene mutations. The distribution of gene mutations did not differ significantly between adults and children (*P* = 0.531). However, the distribution of mutations varied across different types of cardiomyopathies. In adults, patients with ACM had the highest prevalence of pathogenic mutations (17 of 24, 70.8%), while patients with HCM had the fewest (2 of 10, 20.0%) (*P* < 0.001).

In total, we identified 28 pathogenic gene mutations associated with cardiomyopathy, with the distribution shown in Table 4. The *HADHA*, *TNNI3,* and *TNNT2* mutations were predominantly found in the pediatric cohort (*P* < 0.05). After applying Benjamini-Hochberg FDR correction for multiple comparisons, only *TNNT2* remained significantly more prevalent in the pediatric cohort (FDR-adjusted *P* = 0.049). Other genes showing nominal significance in raw comparisons, including *HADHA* (raw *P* = 0.036, FDR-adjusted *P* = 0.126) and *TNNI3* (raw *P* = 0.036, FDR-adjusted *P* = 0.126), did not retain statistical significance after correction (Supplemental Table 7). *MYH7* mutation was the most prevalent in the adult cohort, accounting for 10 of 65 cases (15.4%), and was most commonly observed in patients with RCM (5 of 10, 50%; Supplemental Table 3). The *LMNA* mutation also had a relatively high frequency (9 of 166, 5.4%) and was predominantly found in the adult cohort (*P* = 0.039; Table 4). Of course, most were from patients with DCM (6 of 9, 66.7%; Table 5). Previous reports indicate that DCM associated with *LMNA* mutations is a common inherited cardiomyopathy characterized by systolic dysfunction and arrhythmias, and these mutations are predominantly observed in adult patients with DCM.^28^

**Table 4:** Distribution of Variants in Cardiomyopathy Between Children and Adults

Gene	Adults (n = 166)	Children (n = 76)	*P* Value
*HADHA*	0 (0; 95% CI: 0-2.2)	2 (2.6%; 95% CI: 0.3-9.2)	**0.036**
*LMNA*	9 (5.4%; 95% CI: 2.5-10.0)	0 (0; 95% CI: 0-4.7)	**0.039**
*TNNI3*	0 (0; 95% CI: 0-2.2)	2 (2.6%; 95% CI: 0.3-9.2)	**0.036**
*TNNT2*	3 (1.8%; 95% CI: 0.4-5.2)	7 (9.2%; 95% CI: 3.8-18.1)	**0.007**
*DES*	0 (0; 95% CI: 0-2.2)	1 (1.3%; 95% CI: 0-7.1)	0.688
*DMD*	1 (0.6%; 95% CI: 0-3.3)	0 (0; 95% CI: 0-4.7)	0.385
*DSC2*	0 (0; 95% CI: 0-2.2)	1 (1.3%; 95% CI: 0-7.1)	0.688
*DSG2*	6 (3.6%; 95% CI: 1.3-7.7)	3 (3.9%; 95% CI: 0.8-11.1)	0.832
*DSP*	3 (1.8%; 95% CI: 0.4-5.2)	0 (0; 95% CI: 0-4.7)	0.238
*FLNC*	1 (0.6%; 95% CI: 0-3.3)	2 (2.6%; 95% CI: 0.3-9.2)	0.207
*GLB1*	2 (1.2%; 95% CI: 0.1-4.3)	0 (0; 95% CI: 0-4.7)	0.218
*GNPTG*	0 (0; 95% CI: 0-2.2)	1 (1.3%; 95% CI: 0-7.1)	0.127
*GUSB*	0 (0; 95% CI: 0-2.2)	1 (1.3%; 95% CI: 0-7.1)	0.127
*MYBPC3*	5 (3.0%; 95% CI: 1.0-6.9)	1 (1.3%; 95% CI: 0-7.1)	0.431
*MYH15*	0 (0; 95% CI: 0-2.2)	1 (1.3%; 95% CI: 0-7.1)	0.127
*MYH6*	2 (1.2%; 95% CI: 0.1-4.3)	0 (0; 95% CI: 0-4.7)	0.218
*MYH7*	10 (6.0%; 95% CI: 2.9-10.8)	3 (3.9%; 95% CI: 0.8-11.1)	0.506
*MYLK2*	1 (0.6%; 95% CI: 0-3.3)	0 (0; 95% CI: 0-4.7)	0.498
*MYPN*	1 (0.6%; 95% CI: 0-3.3)	0 (0; 95% CI: 0-4.7)	0.498
*NAGLU*	0 (0; 95% CI: 0-2.2)	1 (1.3%; 95% CI: 0-7.1)	0.127
*PKP2*	2 (1.2%; 95% CI: 0.1-4.3)	1 (1.3%; 95% CI: 0-7.1)	0.942
*PLN*	2 (1.2%; 95% CI: 0.1-4.3)	0 (0; 95% CI: 0-4.7)	0.218
*PRF1*	1 (0.6%; 95% CI: 0-3.3)	0 (0; 95% CI: 0-4.7)	0.498
*RET*	1 (0.6%; 95% CI: 0-3.3)	0 (0; 95% CI: 0-4.7)	0.498
*SCN5A*	1 (0.6%; 95% CI: 0-3.3)	0 (0; 95% CI: 0-4.7)	0.498
*SLC22A5*	1 (0.6%; 95% CI: 0-3.3)	0 (0; 95% CI: 0-4.7)	0.498
*TGFBR2*	1 (0.6%; 95% CI: 0-3.3)	0 (0; 95% CI: 0-4.7)	0.498
*TTN*	12 (7.2%; 95% CI: 3.8-12.3)	3 (3.9%; 95% CI: 0.8-11.1)	0.326
*WNT1*	0 (0; 95% CI: 0-2.2)	1 (1.3%; 95% CI: 0-7.1)	0.127

95% CI for proportions: Wilson score method for n ≥ 30 in the respective groups, Clopper-Pearson exact method for n < 30. Bolded *P* values indicate statistically significant differences between groups.

**Table 5:** Distribution of Variants With DCM Between Children and Adults

Gene	Variants Based on Age Group			Variants Based on Time to HTx (mo)^a^		
	Adults (n = 120)	Children (n = 49)	*P* Value	Adults	Children	*P* Value
*TNNT2*	2 (1.7%; 95% CI: 0.2-5.9)	7 (14.3%; 95% CI: 6.0-27.0)	**0.001**	128 (128-250; 95% CI: 116-250)	19 (8-34; 95% CI: 8-34)	**0.002**
*HADHA*	0 (0; 95% CI: 0-3.0)	2 (4.1%; 95% CI: 0.5-14.0)	**0.026**	—	—	—
*MYH7*	2 (1.7%; 95% CI: 0.2-5.9)	2 (4.1%; 95% CI: 0.5-14.0)	0.349	54 (54-76; 95% CI: 54-76)	32 (3-32; 95% CI: 3-32)	0.301
*TTN*	10 (8.3%; 95% CI: 4.1-14.8)	3 (6.1%; 95% CI: 1.3-16.9)	0.854	5 (2-32; 95% CI: 2-32)	5 (3-32; 95% CI: 3-32)	0.322
*LMNA*	6 (5.1%; 95% CI: 1.9-10.6)	0 (0; 95% CI: 0-7.3)	0.111	—	—	—
*DSP*	2 (1.7%; 95% CI: 0.2-5.9)	0 (0; 95% CI: 0-7.3)	0.363	—	—	—
*PKP2*	0 (0; 95% CI: 0-3.0)	1 (2.0%; 95% CI: 0.1-10.9)	0.117	—	—	—
*DSG2*	0 (0; 95% CI: 0-3.0)	1 (2.0%; 95% CI: 0.1-10.9)	0.117	—	—	—
*GNPTG*	0 (0; 95% CI: 0-3.0)	1 (2.0%; 95% CI: 0.1-10.9)	0.117	—	—	—
*MYH6*	2 (1.7%; 95% CI: 0.2-5.9)	0 (0; 95% CI: 0-7.3)	0.363	—	—	—
*NAGLU*	0 (0; 95% CI: 0-3.0)	1 (2.0%; 95% CI: 0.1-10.9)	0.117	—	—	—
*DMD*	1 (0.8%; 95% CI: 0-4.6)	0 (0; 95% CI: 0-7.3)	0.522	—	—	—
*FLNC*	1 (0.8%; 95% CI: 0-4.6)	0 (0; 95% CI: 0-7.3)	0.522	—	—	—
*GLB1*	2 (1.7%; 95% CI: 0.2-5.9)	0 (0; 95% CI: 0-7.3)	0.363	—	—	—
*MYBPC3*	4 (3.4%; 95% CI: 0.9-8.3)	0 (0; 95% CI: 0-7.3)	0.196	—	—	—
*MYPN*	1 (0.8%; 95% CI: 0-4.6)	0 (0; 95% CI: 0-7.3)	0.522	—	—	—
*RET*	1 (0.8%; 95% CI: 0-4.6)	0 (0; 95% CI: 0-7.3)	0.522	—	—	—
*SCN5A*	1 (0.8%; 95% CI: 0-4.6)	0 (0; 95% CI: 0-7.3)	0.522	—	—	—
*SLC22A5*	1 (0.8%; 95% CI: 0-4.6)	0 (0; 95% CI: 0-7.3)	0.522	—	—	—
*TGFBR2*	1 (0.8%; 95% CI: 0-4.6)	0 (0; 95% CI: 0-7.3)	0.522	—	—	—

95% CI for proportions: Wilson score method for n ≥ 30 in the respective group, Clopper-Pearson exact method for n < 30. Bolded *P* values indicate statistically significant differences between groups.

DCM = dilated cardiomyopathy; HTx = heart transplantation.

a Time to HTx indicates time from initial diagnosis to heart transplantation in months.

Further analysis revealed that *TNNT2* mutations were predominantly found in pediatric versus adult patients with DCM (1.7% [2 of 12] vs 14.3% [7 of 49], *P* = 0.001; Table 5). These mutations were associated with shorter time from diagnosis to HTx compared to adults with DCM (median: 128 vs 19 months; Table 5). Among the 7 unrelated pediatric with *TNNT2* mutation, 4 of 7 (57.1%) carried the same non-frameshift mutation p.K220del, which causes conformational changes at the protein level (Figure 4A). This suggests that the *TNNT2* mutation is an important risk factor for accelerated time from diagnosis to HTx in pediatric DCM. Additionally, we observed that the *HADHA* mutation was predominantly found in pediatric versus adult DCM patients (0% vs 4.1% [2 of 49], *P* = 0.026; Table 5). Children with the *HADHA* mutation showed significantly shorter time to HTx compared to those without the mutation (average time compared to those without the mutation: 5 vs 22 months, *P* = 0.011). In the pediatric HCM subgroup (n = 4), all patients carried pathogenic mutations. *TNNI3* mutations were observed in 2 of the 4 children (50.0%), whereas no *TNNI3* mutations were identified in the adult HCM patients (0 of 10). Because of the small sample size, we applied Firth's penalized logistic regression to assess this difference, which yielded a Firth-corrected *P* value of 0.024 (Supplemental Tables 4 and 8). The time from diagnosis to HTx of children with *TNNI3* mutation was shorter than those without the mutation (average: 13 vs 45 months, *P* = 0.023).

**Figure 4:**
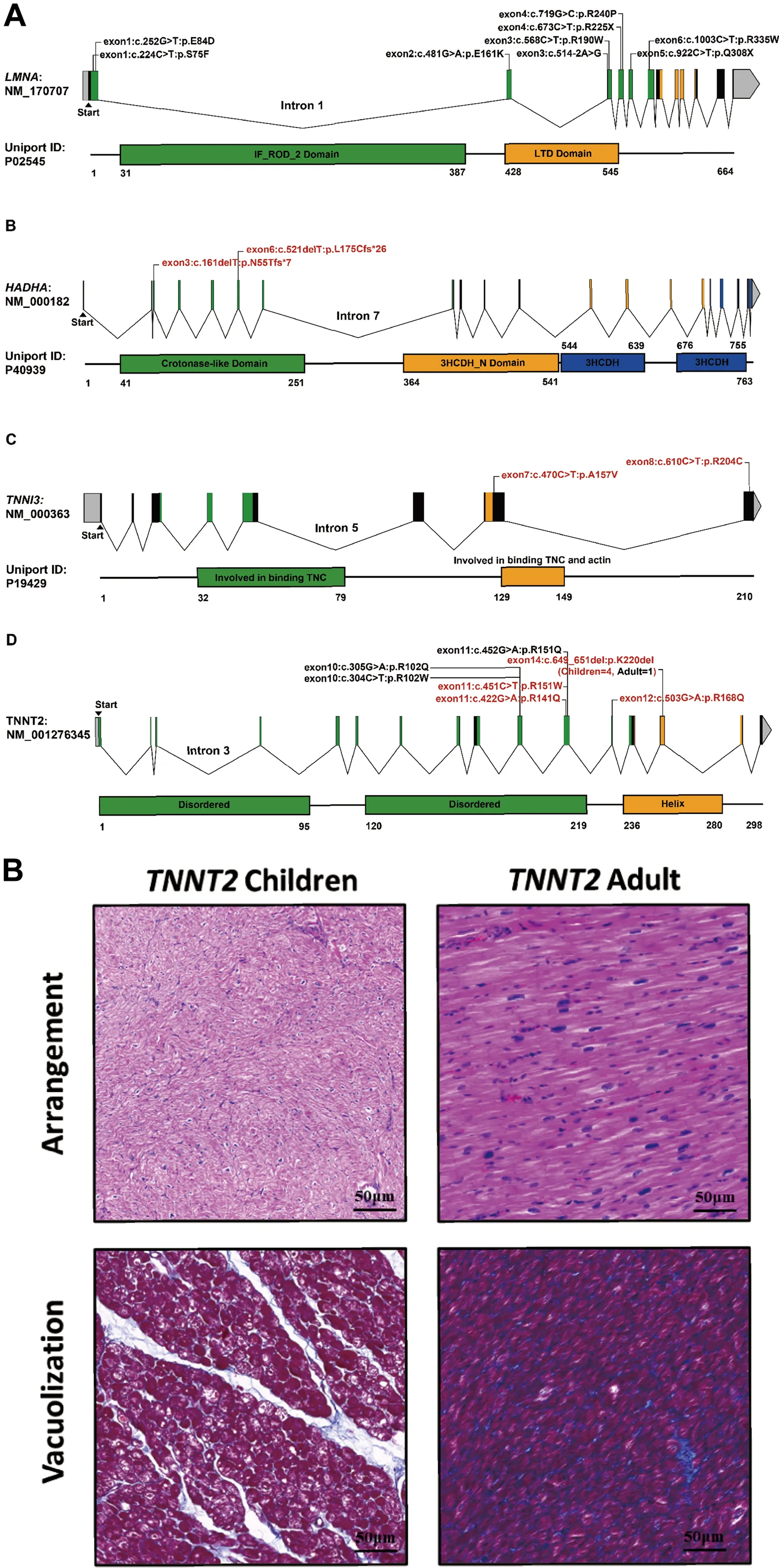
Genetic Mutation Sites and Histopathology of *TNNT2*-Associated Cardiomyopathy (A) Schematic representation of protein domains and the most common mutation site p.K220del in *TNNT2*, along with domain structures of *LMNA*, *HADHA*, and *TNNI3* (not drawn to scale). (B) Representative histology of explanted hearts from children and adults carrying *TNNT2* mutations. Hematoxylin and eosin and Masson's trichrome staining (magnification ×400; scale bar 50 μm). Pediatric myocardium shows more pronounced cardiomyocyte disarray and vacuolization compared to adult tissue. These findings suggest accelerated myocardial remodeling in children with *TNNT2* mutations.

To evaluate the independent prognostic value of *TNNT2* mutations in pediatric DCM patients, we performed a subgroup analysis restricted to children with DCM (n = 49). Patients were stratified by *TNNT2* mutation status (carriers vs noncarriers). In the multivariable accelerated failure time model adjusting for sex, age at diagnosis, baseline LVEF, and LVIDd, *TNNT2* mutation remained an independent predictor of shorter time to HTx (time ratio = 0.38 [95% CI: 0.21-0.69], *P* = 0.002; Table 6). Other independent predictors included baseline LVEF (time ratio = 1.12 per 5% increase [95% CI: 1.02-1.23], *P* = 0.018; Table 6) and baseline LVIDd (time ratio = 0.89 per 5-mm increase [95% CI: 0.81-0.98], *P* = 0.021; Table 6), consistent with expected disease severity markers.

**Table 6:** Multivariable Accelerated Failure Time Model for Time to HTx in Pediatric DCM Patients

	Time Ratio	95% CI	*P* Value
*TNNT2* mutation (carrier vs noncarrier)	0.38	0.21-0.69	**0.002**
Age at diagnosis (per 1-year increase)	1.04	0.98-1.10	0.186
Sex (male vs female)	0.92	0.63-1.34	0.654
Baseline LVEF (per 5% increase)	1.12	1.02-1.23	**0.018**
Baseline LVIDd (per 5-mm increase)	0.89	0.81-0.98	**0.021**

Model based on log-normal distribution. Time ratio <1 indicates shorter time to heart transplantation. Bolded *P* values indicate statistical significance.

DCM = dilated cardiomyopathy; HTx = heart transplantation; LVEF = left ventricular ejection fraction; LVIDd = left ventricular internal diameter at end-diastole.

### Pathologic analysis

In the pediatric cardiomyopathy cohort, Masson's staining revealed that children with *TNNT2* mutation showed a higher presence of unorganized and vacuolated cells compared to adults with the same genetic mutation (Figure 4B and Supplemental Figure 3). Additionally, hematoxylin and eosin staining indicated that the myocardial architecture in children with *TNNT2* mutation was more disorganized than that seen in adults (Figure 4B and Supplemental Figure 3), which is consistent with the previous findings.^29^ The observed cardiomyocyte disorganization and vacuolation may suggest a more rapid progression from DCM to end-stage HF in children.

## Discussion

As studies on childhood cardiomyopathy have progressed, there has been a growing understanding of its genetic underpinnings. Cardiomyopathy in children is a genetically driven disease, where genetic mutations play a pivotal role in its development and progression.^30^ Recent research has emphasized the importance of genetic testing in diagnosing pediatric cardiomyopathy, and the latest guidelines now recommend genetic testing as a key diagnostic tool for this condition.^31^^,^^32^ Additionally, some studies have suggested that genetic factors may contribute to the variability in the prognosis of childhood cardiomyopathy.^33^ In particular, cardiomyopathy is common in children with mitochondrial disease (affecting up to 21% of patients), and it has been associated with increased mortality rates.^34^ Clinically, we have observed that some children with cardiomyopathy had a shorter time from diagnosis to transplantation, while others may not undergo transplantation until adulthood (Tables 1 and 2). In the case of HCM, research has shown that children with this condition have a shorter time from diagnosis to transplantation compared to adults.^12^ Further studies have highlighted significant differences in genetic variants between pediatric and adult patients with end-stage cardiomyopathy, suggesting that genetic factors may contribute to this variability.^35^ However, the role of genetic mutations in children with end-stage cardiomyopathy has not been extensively studied. In this study, we aim to identify and discuss the differences in progression between pediatric and adult patients with end-stage cardiomyopathy (Central Illustration).

**Central Illustration:**
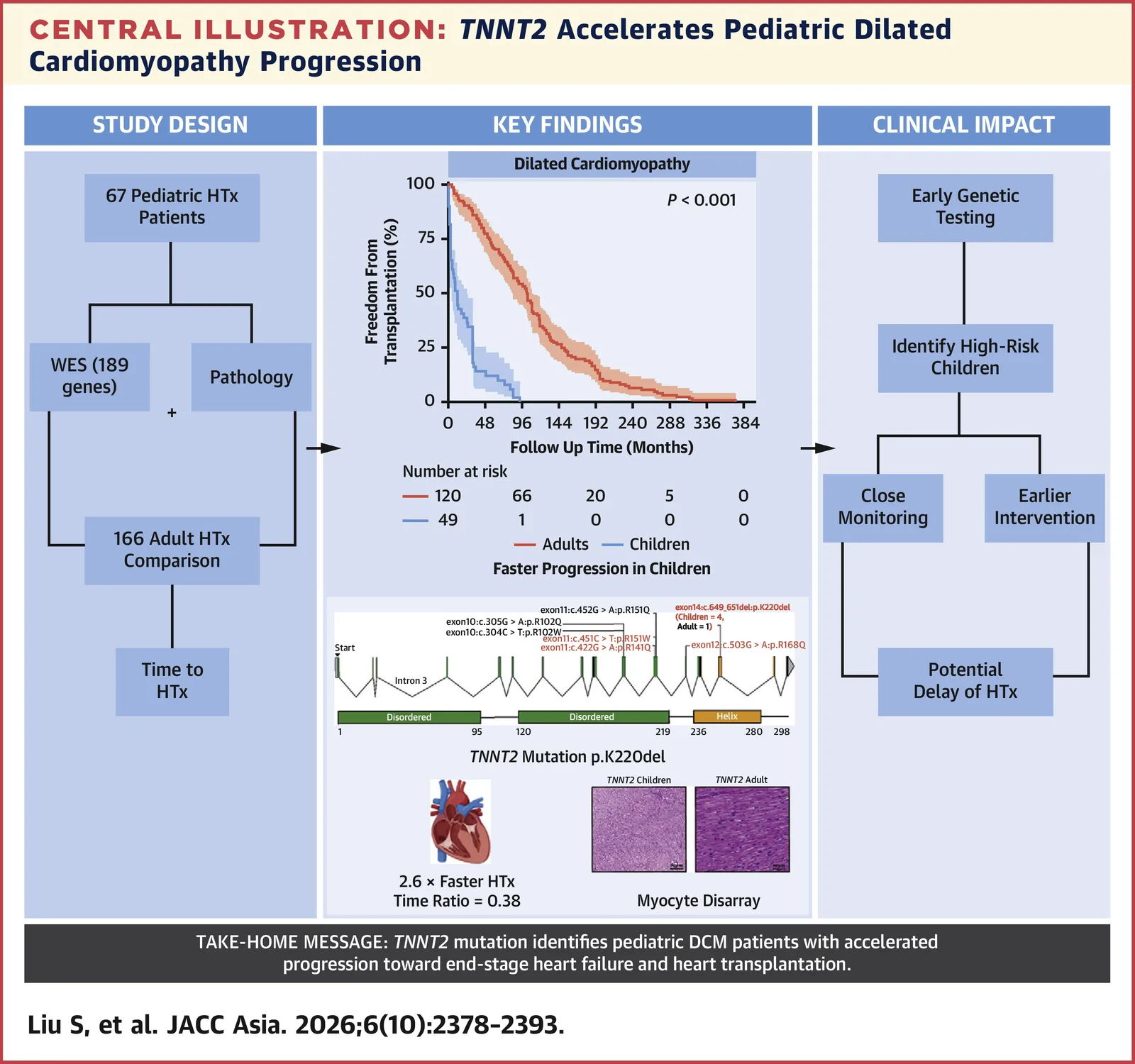
*TNNT2* Accelerates Pediatric Dilated Cardiomyopathy Progression (Left) A total of 67 children with end-stage heart failure in cardiomyopathy underwent heart transplantation (HTx) at our center. Whole exome sequencing (WES) was performed in all patients. Specimens from all patients were analyzed for pathologic staining. (Center and Right) The time from the first diagnosis to transplantation was significantly shorter in children compared to adults in dilated cardiomyopathy, hypertrophic cardiomyopathy and arrhythmogenic cardiomyopathy. In dilated cardiomyopathy (DCM), children were more likely to carry the *TNNT2* mutation (*P* = 0.001), and heart failure progressed more rapidly in children with this mutation (*P* = 0.002). The most common mutation site in these children was p.K220del.

In our pediatric end-stage cardiomyopathy transplantation cohort, we observed significant differences in the distribution of etiologies. DCM was the predominant cause of HTx in children, accounting for % of cases (Figure 2A). This finding is consistent with reports from other centers, where DCM was also the leading etiology for end-stage cardiomyopathy in children.^36^ DCM remains one of the most important indications for HTx in the pediatric population. Furthermore, we found that children with DCM had a shorter time from diagnosis to HTx, with an average time of 12 months (*P* = 0.043; Table 1 and Supplemental Figure 1). These children also exhibited significantly lower LVEF (median: 25%, *P* < 0.001; Table 1 and Figure 2B) and larger LVIDd (median: 65 mm, *P* < 0.001; Table 1 and Figure 2B) compared to those with other types of cardiomyopathies. Previous studies have established that a low ejection fraction is an independent risk factor for poor prognosis in pediatric DCM,^37^ underscoring the shorter time from diagnosis to HTx in this cohort. When comparing the time from diagnosis to HTx, children with DCM (median: 12 vs 103 months, *P* < 0.001; Tables 1 and 2 and Figure 3A), HCM (median: 32 vs 234 months, *P* = 0.002; Tables 1 and 2 and Figure 3B), and ACM (median: 14 vs 43 months, *P* < 0.001; Tables 1 and 2 and Figure 3D) all showed significantly shorter times than adults. Overall, the time to HTx in pediatric cardiomyopathy was notably shorter than that in the adult population.

In addition, in terms of the proportion of genetic mutations, we found that all children with HCM and RCM carried genetic mutations (*P* = 0.03; Table 1 and Figure 2B). Genetic mutations are known to be a major causative factor in pediatric cardiomyopathy. When compared with the adult cohort, the proportion of mutations in pediatric HCM was significantly higher than in adults (20.0% vs 100.0%, *P* = 0.015). Previous studies have shown that patients with HCM who present in childhood are more likely to carry pathogenic mutations and are at an increased risk for HF later in life.^12^ Although there were no significant differences in the proportion of genetic mutations between DCM, RCM, and ACM (Table 3), we observed significant differences in the types of mutations. For example, *TNNT2* was the most frequent mutation in childhood DCM (14.3%, *P* = 0.026). Correlation analysis of the association between the time from diagnosis to HTx showed that children with the *TNNT2* mutation in DCM had a shorter time to HTx (median: 128 vs 19 months, *P* = 0.002), and most (57.1%) of these cases involved the same non-frameshift mutation p.K220del. *TNNT2* encodes sarcomere and is associated with a worse prognosis.^33^ *TNNT2* variants cause reduced cardiac microtissue contraction, leading to a decline in cardiac function decreasing. Related studies have found that *TNNT2* mutation reduces myofilament-directed calcium transients. This in turn alters the calcium affinity of the filaments, which leads to corresponding changes in filament activation and force generation.^29^ The mutation also increases myofilament disassembly. We observed a more disturbed arrangement of cardiomyocytes in pathological staining of children with *TNNT2* mutation (Figure 4B).

In addition, we identified the *HADHA* mutation in DCM, which affects energy metabolism and contributes to myocardial fibrosis.^38^^,^^39^ In HCM, we found that children were more likely to carry the *TNNI3* mutation (0% vs 50.0%, *P* = 0.016; Supplemental Table 4). *TNNI3* is a gene that encodes sarcomere proteins and is associated with poor prognosis.^33^ Our cohort showed that children carrying the *TNNI3* mutation also had a shorter time from diagnosis to HTx (median: 13 vs 45 months, *P* = 0.023). These genetic mutations may also represent potential risk factors that warrant further investigation.

### Study Limitations

Firstly, it is important to note that this study is a retrospective cohort observational study, which may affect the persuasiveness of the conclusions because of the limitations of the total cohort size. Nevertheless, it is worth mentioning that this study focused on a pediatric HTx cohort with end-stage cardiomyopathy that had complete genetic and clinical information and that were compared with an adult cardiomyopathy cohort. Secondly, given the retrospective design, there may be a risk of selection bias. Moreover, the influence of family genetic background remains an important factor that requires further investigation, especially for some mutated genes associated with fast-progressing childhood cardiomyopathies. Because of the inherent differences between the pediatric and adult cohorts in terms of age, comorbidities, and medication history, the comparative results between the 2 groups have certain limitations. Finally, external environmental factors are also potential risk factors for disease progression, and they should not be ignored, requiring further research.

## Conclusions

Cardiomyopathy in children is genetically heterogeneous, and we have observed that the time from diagnosis to transplantation tends to be faster in pediatric patients. Genetic analyses further indicate that the *TNNT2* mutation is particularly associated with an increased risk of shorter time to transplantation. Given this, early genetic testing is crucial for pediatric cardiomyopathy patients, as it can help identify those at high risk. Children identified as being at high risk should receive more intensive monitoring and treatment strategies, aiming to reduce the time or even prevent the onset of transplantation. This proactive approach is expected to significantly improve the overall prognosis of pediatric cardiomyopathy.

### Data Availability

The data underlying this report will be shared on reasonable request to the corresponding author.

### Ethics Statement

The study protocol and data publication were approved by the Institutional Review Board (IRB) of Fuwai Hospital according to the Declaration of Helsinki (approval number: 2022-1727, approval date: October 13, 2022).

## Funding Support and Author Disclosures

This work is partially supported by the National Natural Science Fund for Distinguished Young Scholars of China (82125004; to Dr Song) and Frontier Biotechnology Key Project of National Key R & D Program of the Ministry of Science and Technology of China (2023YFC2706204; to Dr Chen). The National Natural Science Foundation of China (Grant No. 82300397). CAMS Innovation Fund for Medical Sciences (CIFMS) [2025-I2M-XHXX-029]. Chinese Academy of Medical Sciences for CAMS Innovation Fund for Medical Sciences (Grant No.2021-I2M-1-064). The authors have reported that they have no relationships relevant to the contents of this paper to disclose.
