# Supplementary material for: TNNT2 Mutation Affects the Progression of Dilated Cardiomyopathy in Children
Source: JACC Asia. 2026 Jul 23;6(10):2378–93. doi: 10.1016/j.jacasi.2026.05.038 (PMC13654378; doi:10.1016/j.jacasi.2026.05.038)
Supplement: Supplementary file 1 [file mmc1.docx]

**Supplemental Tables**

**Table S1**

**Gene Symbol**

*ACADM, ACTA2, ACTC1, ACTN2, ACVRL1, AGA, AGK, AGL, AGXT, ANKRD1, APOB, ARSB, BMPR1B, BMPR2, BRAF, CACNAIC, CALM1, CALM2, CALR3, CASQ2, CAV1, CAV3, CBL, CDH2, CLCN2, CNS, COA5, COL3A1, COX15, CPT1A, CPT2, CSRP3, CTNNA3, CTP17A1, CTSA, CYP11B1, CYP11B2, DES, DMD, DSC2, DSG2, DSP, DTNA, EIF2AK4, ENG, EPAS1, ETFA, ETFB, ETFDH, F2, F5, FAH, FBN1, FH, FHL1, FLNC, FXN, GAA, GALNS, GBA, GBE1, GLA, GLB1, GNB2, GNPTAB, GNPTG, GNS, GRHPR, GUL3, GUSB, GYS1, GYS1 , HADHA, HCN4, HEXB, HGSNAT, HOGA1, HSD11B2, IDS, IDUA, JPH2, JUP, KCNE1, KCNH2, KCNJ2, KCNJ5, KCNK3, KCNQ1, KLHL3, KRAS, LAMP2, LDB3, LDB3 , LDLR, LDLRAP1, LMNA, LOX, MAP2K1, MAP2K2, MAX, MLYCD, MTCO1, MTCYB, MTND4, MTRNR2, MTTK, MTTL1, MYBPC3, MYH11, MYH6, MYH7, MYL2, MYL3, MYLK, MYLK2, MYOZ2, MYPN, NAGA, NAGLU, NDUFS2, NDUFV2, NEXN, NF1, NKX2-5, NR3C1, PCCA, PCCB, PCSK9, PHKA1, PHKA2, PHKB, PHKG2, PHYH, PKP2, PLN, PRKAG2, PRKG1, PROC, PROS1, PTPN11, PUN, RAF1, RBM20, RCM2, RET, RYR2, SCN1B, SCN5A, SCNN1B, SCNN1G, SCNSA, SCO2, SDHA, SDHAF2, SDHB, SDHC, SDHD, SERPINC1, SGSH, SKI, SLC22A5, SLC25A20, SLC25A4, SMAD3, SMAD9, SOS1, SURF1, TAZ, TCAP, TGFB2, TGFB3, TGFBR1, TGFBR2, TIN, TMEM127, TMEM43, TNN13, TNNC1, TNNI3, TNNT2, TPM1, TRPM4, TTN, TTR, VCL, VHL, VLCAD, WNK1, WNK4*

**Table S2: Detailed Information of Pathogenic and Likely Pathogenic (P/LP) Variants Identified in the Adult and Pediatric Cohorts.**

| **Gene** | **Transcript)** | **Exon** | **Nucleotide** | **Protein** | **Type** | **ACMG** | **Freq** | **Adults** | **Children** |
| --- | --- | --- | --- | --- | --- | --- | --- | --- | --- |
| *DES* | NM_001382709 | exon6 | c.C928T | p.R310W | Missense | P | Absent | 0 | 1 |
| *DMD* | NM_000109 | exon41 | c.5716-2A>T | Splicing | Splicing | P | Absent | 1 | 0 |
| *DSC2* | NM_004949 | exon14 | c.C2219A | p.S740X | Nonsense | P | Absent | 0 | 1 |
| *DSG2* | NM_001943 | exon11 | c.T1592G | p.F531C | Missense | LP | GnomAD: 0.0013 | 2 | 3 |
| *DSG2* | NM_001943 | exon6 | c.A593G | p.Y198C | Missense | LP | Absent | 0 | 1 |
| *DSG2* | NM_001943 | exon11 | c.G1525A | p.D509N | Missense | LP | Absent | 1 | 0 |
| *DSG2* | NM_001943 | exon13 | c.T1952C | p.I651T | Missense | LP | Absent | 1 | 0 |
| *DSG2* | NM_001943 | exon3 | c.C136T | p.R46W | Missense | LP | Absent | 1 | 0 |
| *DSG2* | NM_001943 | exon5 | c.C425T | p.P142L | Missense | LP | Absent | 1 | 0 |
| *DSP* | NM_001319034 | exon23 | c.C3799T | p.R1267X | Nonsense | P | Absent | 1 | 0 |
| *DSP* | NM_001319034 | exon4 | c.597+1G>A | Splicing | Splicing | LP | Absent | 1 | 0 |
| *DSP* | NM_001008844 | exon24 | c.C6247G | p.Q2083E | Missense | LP | GnomAD: 0.0006 | 1 | 0 |
| *FLNC* | NM_001127487 | exon32 | c.5347_5348del | p.D1783Qfs*14 | Frameshift | LP | Absent | 1 | 0 |
| *FLNC* | NM_001127487 | exon21 | c.C3557T | p.A1186V | Missense | LP | Absent | 0 | 2 |
| *GLB1* | NM_001317040 | exon2 | c.C79T | p.R27X | Nonsense | P | 1000g: 0.001 | 1 | 0 |
| *GLB1* | NM_000404 | exon6 | c.C716T | p.T239M | Missense | LP | Absent | 1 | 0 |
| *GNPTG* | NM_032520 | exon11 | c.867_880del | p.K290Afs*4 | Frameshift | P | Absent | 0 | 1 |
| *GUSB* | NM_001284290 | exon5 | c.G775A | p.D259N | Missense | LP | GnomAD: 0.001 | 0 | 1 |
| *HADHA* | NM_000182 | exon3 | c.161delT | p.N55Tfs*7 | Frameshift | P | Absent | 0 | 1 |
| *HADHA* | NM_000182 | exon6 | c.521delT | p.L175Cfs*26 | Frameshift | P | Absent | 0 | 1 |
| *LMNA* | NM_170707 | exon1 | c.C224T | p.S75F | Missense | LP | Absent | 1 | 0 |
| *LMNA* | NM_170707 | exon1 | c.G252T | p.E84D | Missense | LP | Absent | 1 | 0 |
| *LMNA* | NM_170707 | exon2 | c.G481A | p.E161K | Missense | LP | Absent | 1 | 0 |
| *LMNA* | NM_170707 | exon3 | c.C568T | p.R190W | Missense | P | Absent | 1 | 0 |
| *LMNA* | NM_170707 | exon3 | c.514-2A>G | - | Splicing | LP | Absent | 1 | 0 |
| *LMNA* | NM_170707 | exon4 | c.G719C | p.R240P | Missense | LP | Absent | 1 | 0 |
| *LMNA* | NM_170707 | exon4 | c.C673T | p.R225X | Nonsense | P | Absent | 1 | 0 |
| *LMNA* | NM_170707 | exon5 | c.C922T | p.Q308X | Nonsense | P | Absent | 1 | 0 |
| *LMNA* | NM_170707 | exon6 | c.C1003T | p.R335W | Missense | P | Absent | 1 | 0 |
| *MYBPC3* | NM_000256 | exon12 | c.G1000A | p.E334K | Missense | P | GnomAD: 0.0026 | 3 | 0 |
| *MYBPC3* | NM_000256 | exon6 | c.G772A | p.E258K | Missense | LP | GnomAD: 6.37e-05 | 1 | 0 |
| *MYBPC3* | NM_000256 | exon31 | c.3624delC | p.K1209Sfs*28 | Frameshift | LP | GnomAD: 4.1e-06 | 1 | 0 |
| *MYBPC3* | NM_000256 | exon18 | c.1827dupC | p.D610Rfs*4 | Frameshift | P | Absent | 0 | 1 |
| *MYH6* | NM_002471 | exon30 | c.4186delG | p.A1396Pfs*32 | Frameshift | LP | Absent | 2 | 0 |
| *MYH7* | NM_000257 | exon13 | c.G1208A | p.R403Q | Missense | LP | Absent | 1 | 1 |
| *MYH7* | NM_000257 | exon19 | c.G2156A | p.R719Q | Missense | P | Absent | 1 | 0 |
| *MYH7* | NM_000257 | exon9 | c.G746A | p.R249Q | Missense | P | Absent | 1 | 0 |
| *MYH7* | NM_000257 | exon5 | c.C449G | p.A150G | Missense | LP | Absent | 1 | 0 |
| *MYH7* | NM_000257 | exon38 | c.C5561T | p.T1854M | Missense | LP | GnomAD: 6.37e-05 | 1 | 0 |
| *MYH7* | NM_000257 | exon30 | c.A4124G | p.Y1375C | Missense | LP | Absent | 1 | 0 |
| *MYH7* | NM_000257 | exon21 | c.G2348A | p.R783H | Missense | LP | GnomAD: 6.37e-05 | 1 | 0 |
| *MYH7* | NM_000257 | exon27 | c.G3669T | p.E1223D | Missense | LP | Absent | 1 | 0 |
| *MYH7* | NM_000257 | exon28 | c.G3801C | p.Q1267H | Missense | LP | 1000g: 0.002 | 1 | 0 |
| *MYH7* | NM_000257 | exon15 | c.A1490G | p.E497G | Missense | LP | Absent | 1 | 0 |
| *MYH7* | NM_000257 | exon31 | c.T4190C | p.L1397P | Missense | LP | Absent | 0 | 2 |
| *MYH7* | NM_000257 | exon15 | c.A1508C | p.K503T | Missense | LP | Absent | 0 | 1 |
| *MYPN* | NM_032578 | exon17 | c.C3481A | p.L1161I | Missense | P | GnomAD: 0.0052 | 1 | 0 |
| *NAGLU* | NM_000263 | exon1 | c.136delG | p.A46Pfs*76 | Frameshift | P | Absent | 0 | 1 |
| *PKP2* | NM_001005242 | exon8 | c.C1819T | p.R607X | Nonsense | P | GnomAD: 5.3e-05 | 1 | 0 |
| *PKP2* | NM_001005242 | exon10 | c.2096delC | p.P699Qfs*3 | Frameshift | P | Absent | 1 | 0 |
| *PKP2* | NM_001005242 | exon2 | c.273_274insGATT | p.L92Dfs*4 | Frameshift | P | Absent | 0 | 1 |
| *PLN* | NM_002667 | exon2 | c.36_38del | p.R14del | In-frame del | P | GnomAD: 3.18e-05 | 2 | 0 |
| *RET* | NM_001355216 | exon10 | c.A1580G | p.Q527R | Missense | LP | 1000g: 0.0002 | 1 | 0 |
| *SCN5A* | NM_001160161 | exon22 | c.C3908T | p.A1303V | Missense | LP | GnomAD: 0.0001 | 1 | 0 |
| *SLC22A5* | NM_003060 | exon8 | c.C1400G | p.S467C | Missense | LP | GnomAD: 0.0021 | 1 | 0 |
| *TGFBR2* | NM_003242 | exon3 | c.383dupA | p.P129Afs*3 | Frameshift | LP | Absent | 1 | 0 |
| *TNNI3* | NM_000363 | exon7 | c.C470T | p.A157V | Missense | LP | Absent | 0 | 1 |
| *TNNI3* | NM_000363 | exon8 | c.C610T | p.R204C | Missense | LP | Absent | 0 | 1 |
| *TNNT2* | NM_001276345 | exon11 | c.G452A | p.R151Q | Missense | P | Absent | 1 | 0 |
| *TNNT2* | NM_001276345 | exon10 | c.G305A | p.R102Q | Missense | P | Absent | 1 | 0 |
| *TNNT2* | NM_001276345 | exon10 | c.C304T | p.R102W | Missense | P | Absent | 1 | 0 |
| *TNNT2* | NM_001276345 | exon11 | c.C451T | p.R151W | Missense | P | Absent | 0 | 1 |
| *TNNT2* | NM_001276345 | exon11 | c.G422A | p.R141Q | Missense | P | Absent | 0 | 1 |
| *TNNT2* | NM_001276345 | exon14 | c.649_651del | p.K220del | In-frame del | LP | Absent | 1 | 4 |
| *TNNT2* | NM_001276345 | exon12 | c.G503A | p.R168Q | Missense | LP | Absent | 0 | 1 |
| *TTN* | NM_133379 | exon46 | c.C12949T | p.Q4317X | Nonsense | P | GnomAD: 0.0001 | 2 | 0 |
| *TTN* | NM_003319 | exon92 | c.22360dupA | p.Y7456Ifs*8 | Frameshift | LP | Absent | 2 | 0 |
| *TTN* | NM_003319 | exon136 | c.36979_36980insT | p.R12327Lfs*2 | Frameshift | LP | Absent | 2 | 0 |
| *TTN* | NM_003319 | exon45 | c.G12853T | p.E4285X | Nonsense | P | Absent | 1 | 0 |
| *TTN* | NM_003319 | exon37 | c.C8904A | p.C2968X | Nonsense | P | Absent | 1 | 0 |
| *TTN* | NM_003319 | exon186 | c.C75366A | p.Y25122X | Nonsense | LP | Absent | 1 | 0 |
| *TTN* | NM_003319 | exon154 | c.T46602G | p.Y15534X | Nonsense | P | Absent | 1 | 0 |
| *TTN* | NM_003319 | exon154 | c.C50583G | p.Y16861X | Nonsense | LP | Absent | 1 | 0 |
| *TTN* | NM_001256850 | exon11 | c.1800+1G>A | Splicing | Splicing | P | GnomAD: 0.0019 | 1 | 0 |
| *TTN* | NM_003319 | exon137 | c.C37258T | p.R12420X | Nonsense | LP | Absent | 0 | 1 |
| *TTN* | NM_003319 | exon155 | c.C59845T | p.R19949X | Nonsense | P | Absent | 0 | 1 |
| *TTN* | NM_003319 | exon186 | c.78515delC | p.P26172Qfs*5 | Frameshift | P | Absent | 0 | 1 |

Abbreviations: P, pathogenic; LP, likely pathogenic; ACMG, American College of Medical Genetics and Genomics; 1000g, 1000 Genomes Project; GnomAD, Genome Aggregation Database. Allele frequencies were obtained from the 1000 Genomes Project and GnomAD databases. "Absent" indicates the variant was not found in the respective databases.

**Table S3: Distribution of Variants With RCM**

| **Gene** | **Variants based on age** | | | **Variants based on Time to HTx(months)** | | |
| --- | --- | --- | --- | --- | --- | --- |
|  | **Adults(n=10)** | **Children(n=3)** | ***P-*value** | **Adults** | **Children** | ***P-*value** |
| ***MYH7*** | 5(50.0%) | 0 | 0.118 | - | - | - |
| ***TNNT2*** | 1(10.0%) | 0 | 0.569 | - | - | - |
| ***TTN*** | 2(20.0%) | 0 | 0.400 | - | - | - |
| ***DES*** | 0 | 1(33.3%) | 0.057 | - | - | - |
| ***GUSB*** | 0 | 1(33.3%) | 0.057 | - | - | - |
| ***DSG2*** | 0 | 1(33.3%) | 0.057 | - | - | - |

RCM, restrictive cardiomyopathy. HTx: heart transplantation.

**Table S4: Distribution of Variants With HCM**

| **Gene** | **Variants based on age** | | | **Variants based on Time to HTx(months)** | | |
| --- | --- | --- | --- | --- | --- | --- |
|  | **Adults(n=10)** | **Children(n=4)** | ***P-*value** | **Adults** | **Children** | ***P-*value** |
| ***TNNI3*** | 0(0) | 2(50.0%) | **0.016** | - | - | - |
| ***MYH7*** | 1(10.0%) | 1(25.0%) | 0.469 | - | - | **-** |
| ***FLNC*** | 0(0) | 1(25.0%) | 0.101 | - | - | - |
| ***MYBPC3*** | 1(10.0%) | 0(0) | 0.512 | - | - | - |

HCM, hypertrophic cardiomyopathy. HTx: heart transplantation.

**Table S5: Distribution of Variants With ACM**

| **Gene** | **Variants based on age** | | | **Variants based on Time to HTx(months)** | | |
| --- | --- | --- | --- | --- | --- | --- |
|  | **Adults(n=24)** | **Children(n=11)** | ***P-*value** | **Adults** | **Children** | ***P-*value** |
| ***DSG2*** | 6(25.0%) | 2(18.2%) | 0.656 | 78(2-186) | 88(88-92) | 0.119 |
| ***PKP2*** | 2(8.3%) | 1(9.1%) | 0.941 | - | - | - |
| ***DSC2*** | 0(0) | 1(9.1%) | 0.134 | - | - | - |
| ***MYBPC3*** | 0(0) | 1(9.1%) | 0.134 | - | - | - |
| ***DSP*** | 1(4.2%) | 0(0) | 0.492 | - | - | - |
| ***LMNA*** | 3(12.5%) | 0(0) | 0.220 | - | - | - |
| ***MYH7*** | 2(8.3%) | 0(0) | 0.324 | - | - | - |
| ***PLN*** | 2(8.3%) | 0(0) | 0.324 | - | - | - |
| ***TNNT2*** | 1(4.2%) | 0(0) | 0.492 | - | - | - |

ACM, arrhythmogenic cardiomyopathy. HTx: heart transplantation.

**Table S6. Clinical Characteristics of *TNNT2* mutation and without *TNNT2* mutation in Pediatric DCM Patients.**

| **Variable** | ***TNNT2* mutation (n=7)** | **Without *TNNT2* mutation(n=42)** | ***P*-value** |
| --- | --- | --- | --- |
| **Age at diagnosis** (year) | 4.2 (1.5-8.0) | 6.8 (2.3-12.1) | 0.186 |
| **Sex** (male, %) | 5 (71.4%) | 27 (64.3%) | 0.714 |
| **Baseline LVEF** (%) | 22.3 ± 6.8 | 24.1 ± 7.2 | 0.523 |
| **Baseline LVIDd** (mm) | 68.2 ± 9.4 | 64.7 ± 8.9 | 0.347 |
| **ICD** (%) | 1 (14.3%) | 1 (2.4%) | 0.122 |
| **VAD** (%) | 0 (0%) | 0 (0%) | 1.000 |
| **Time from diagnosis to HTx** (month) | 19 (8-34) | 45 (22-78) | **0.008** |

Data are expressed as median (IQR), mean ± standard deviation, or n (%).DCM, dilated cardiomyopathy; ICD, implantable cardioverter defibrillator; LVEF, left ventricular ejection fraction; LVIDd, left ventricular internal dimension in diastole; VAD, ventricular assist device; HTx: heart transplantation.

**Table S7. Benjamini-Hochberg FDR-corrected *P*-value.**

| **Gene** | **Raw *P*-value** | | **FDR-corrected *P*-value** | | **Is it still significant (FDR < 0.05)?** |
| --- | --- | --- | --- | --- | --- |
| ***TNNT2*** | | **0.007** | | **0.049** | **Yes** |
| ***HADHA*** | | **0.036** | | 0.126 | No |
| ***TNNI3*** | | **0.036** | | 0.126 | No |
| ***LMNA*** | | **0.039** | | 0.126 | No |

**Table S8. Firth's Penalized Logistic Regression for Rare Variant Comparisons**

| **Comparison** | **Standard P-value** | **Firth-corrected P-value** | **Conclusion** |
| --- | --- | --- | --- |
| **HCM: *TNNI3* (pediatric vs adult)** | 0.016 | 0.024 | Significant |
| **RCM: Overall mutation rate** | NA | NA^*^ | Not applicable |
| ***HADHA* in DCM (pediatric vs adult)** | 0.026 | 0.031 | Significant |
| ***TTN* truncating variants in DCM** | 0.322 | 0.338 | No Significant |

*All 3 pediatric RCM patients carried mutations, but no statistical comparison was performed due to small sample size; results are descriptive only.
**Supplemental Figures**

**Figure S1: K-M curves for time to freedom from heart transplantation in children with different** **dieases.**

ACM, arrhythmogenic cardiomyopathy; DCM, dilated cardiomyopathy; RCM, restrictive cardiomyopathy; HCM, hypertrophic cardiomyopathy.

**Figure S2: K-M curves for time to freedom from heart transplantation in adults with different types of cardiomyopathies.**

ACM, arrhythmogenic cardiomyopathy; DCM, dilated cardiomyopathy; RCM, restrictive cardiomyopathy; HCM, hypertrophic cardiomyopathy.

**Figure S3. Quantitative Histopathologic Analysis of Explanted Hearts from *TNNT2* Mutation Carriers.**
